# Severe Hemolytic Anemia After Mothball Ingestion Complicated by an Undiagnosed Enzyme Deficiency: A Case Report

**DOI:** 10.7759/cureus.48733

**Published:** 2023-11-13

**Authors:** Zainab Mehdi, Monica Gupta, Jasjit Kaur, Isha Sharma, Sahil Mann

**Affiliations:** 1 General Medicine/Emergency Medicine, Government Medical College and Hospital, Chandigarh, Chandigarh, IND; 2 Internal Medicine, Government Medical College and Hospital, Chandigarh, Chandigarh, IND; 3 Psychiatry, Guru Gobind Singh Medical College, Faridkot, IND; 4 General Medicine, Government Medical College and Hospital, Chandigarh, Chandigarh, IND

**Keywords:** methemoglobinemia, hemolytic anemia, naphthalene toxicity, glucose 6 phosphatase deficiency, mothballs

## Abstract

Ingestion of mothballs containing naphthalene or paradichlorobenzene is known to cause hemolysis and methemoglobinemia secondary to severe oxidative stress, affecting the oxygen delivery system of the body. The gradual accumulation of oxidizing radicals in the setting of restricted glutathione availability leads to the oxidization of hemoglobin and other body proteins, ultimately causing cell destruction. In the setting of glucose-6-phosphate dehydrogenase deficiency (G6PDD), more pronounced symptoms and poor prognosis are anticipated as adequate nicotinamide adenine dinucleotide phosphate is not generated to protect red blood cells from oxidative injury, potentiating the hemolytic process further. Here, we report the case of a young male with mothball ingestion whose presentation and management were complicated by underlying undiagnosed G6PDD.

## Introduction

In 2018, the United States alone recorded more than 1,000 cases of mothball intoxication [1]. The vulnerable population comprises children less than five years of age who often present with accidental ingestion of these household products. The main component found in mothballs is naphthalene, an aromatic, lipophilic hydrocarbon that gets metabolized to α-naphthol, β-naphthol, α-naphthoquinone, and β-naphthoquinone in the human body. A lesser toxic ingredient is paradichlorobenzene (PDCB) [2,3]. Both compounds are notorious for causing hemolysis by inducing oxidative stress upon intoxication. The metabolites overwhelm the glutathione stores causing oxidation of hemoglobin and ultimately red blood cell (RBC) lysis. Starvation augments the release of PDCB in the blood owing to the mobilization of fatty reserves in reaction to stress, resulting in a sustained increase in serum levels even after cessation of exposure to the hydrocarbon compound, which is known as Coasting [4-6].

The hallmark of naphthalene intoxication is hypoxia that does not respond to oxygen supplementation along with gastrointestinal symptoms, fever, and headache. Complications such as hepatic dysfunction, seizures and cerebral edema, rhabdomyolysis and renal failure, respiratory insufficiency, and methemoglobinemia have been reported and vary according to the severity of exposure [7]. Naphthalene toxicity is more marked and associated with increased morbidity and mortality in patients with glucose-6-phosphate dehydrogenase deficiency (G6PDD). Increased severity of symptoms can be seen in patients with G6PDD, i.e., severe hemolysis requiring multiple transfusions. Therefore, a high index of suspicion is critical in such cases so that timely ascorbic acid or N-acetylcysteine (NAC) is administered. Methylene blue is commonly used to treat methemoglobinemia but is relatively contraindicated in patients with G6PD deficiency due to the threat of aggravating hemolysis [3,4]. Here, we report the case of a young male with naphthalene toxicity with severe hemolytic anemia and methemoglobinemia due to exaggerated oxidative stress resulting from concomitant G6PD deficiency.

## Case presentation

An 18-year-old healthy young male was brought to emergency medicine by his sister with the chief complaint of pain in the abdomen and loose stools. He was alleged to have ingested four mothballs 48 hours before the admission. The pain was generalized, not limited to any specific quadrant, and was associated with one episode of non-projectile, non-bilious vomiting and six episodes of watery, non-bloody diarrhea with high-colored urine. There was no history of self-harm, attempted suicide, or ongoing treatment for any psychiatric illnesses. He was never hospitalized previously, was not taking any medications, and denied the use of any illicit substances or alcohol.

On examination, the patient was conscious with a Glasgow Coma Score score of 15/15. He appeared pale, jaundiced, and in physical distress. His blood pressure was 140/90 mmHg, pulse rate was 110 beats per minute, respiratory rate was 40 beats per minute, oxygen saturation (SpO_2_) was 75% on room air, and random blood sugar was 97 mg/dL. His chest was clear on auscultation without any crepitations or wheezes, his abdomen was non-tender, without organomegaly, and he had normal bowel sounds. There were no signs of any physical injury to the body.

**Figure 1:**
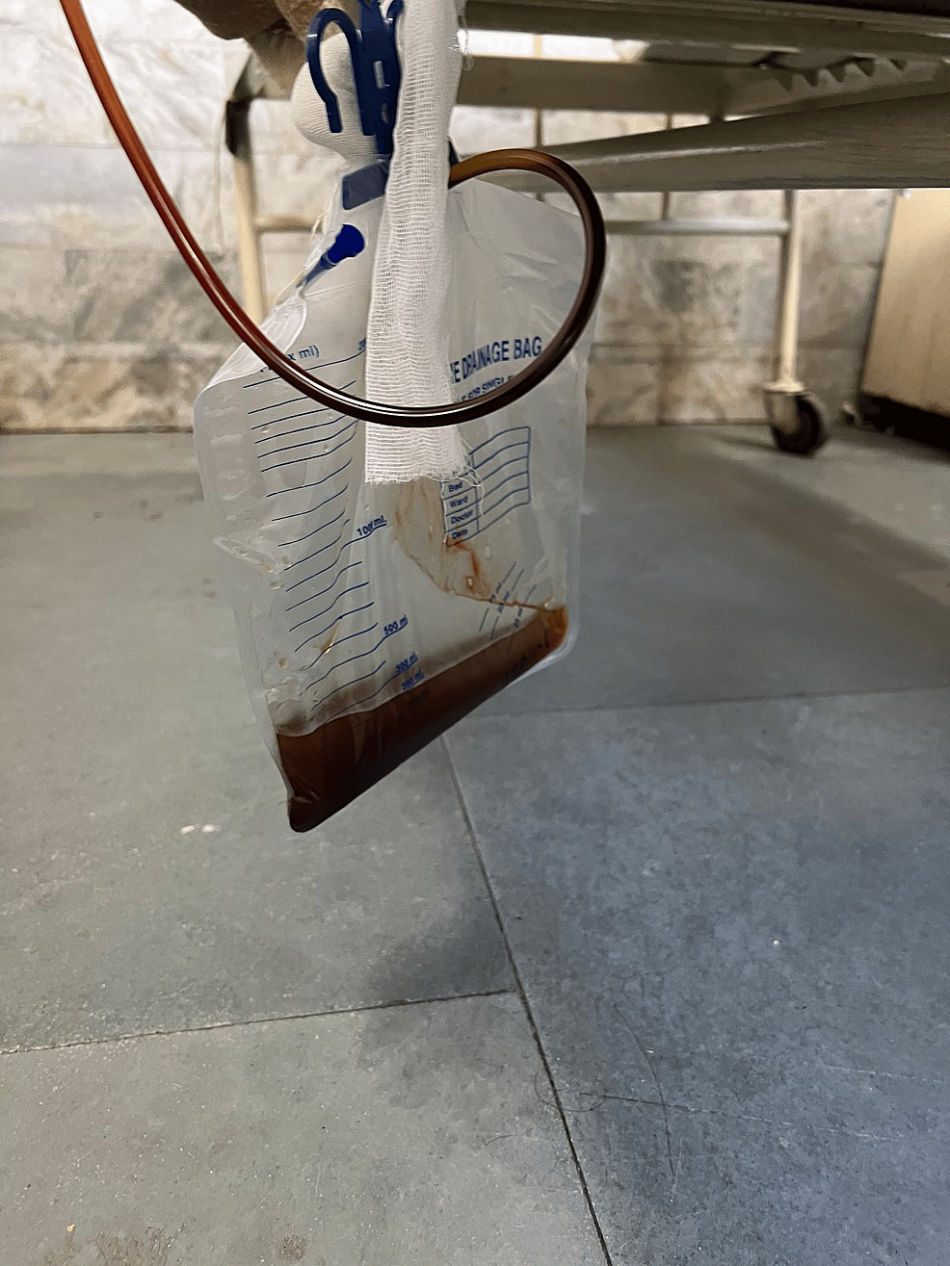
Urine collection bag showing cola-colored urine.

He was immediately admitted to the medical intensive care unit for hemodynamic monitoring and was administered high-flow oxygen via a non-rebreather mask. A Foley catheter was inserted which revealed high-colored urine (Figure 1).

A primary blood and radiological workup was done alongside arterial blood gas analysis and methemoglobin (MetHb) levels. His hypoxia and cyanosis did not show any improvement upon oxygen supplementation and he remained tachypneic. Complete blood count (CBC) revealed moderate anemia with reticulocyte count >3%, mild anisopoikilocytosis, normocytes, and macro-ovalocytes, PaO_2_ was within normal limits with a MetHb level of >30%, and hyperbilirubinemia. Mild transaminitis was seen but a coagulation profile was normal as were the renal function parameters. His urine hemoglobin was 647.5 mg/dL, and plasma hemoglobin was 701.6 mg/dL, supporting hemolytic anemia (Table 1).

**Table 1:** A summary of laboratory parameters of the patient during the hospital stay. MCV = mean corpuscular volume; PCV = packed cell volume; Na = serum sodium; K = serum potassium; TLC = total leucocyte count; ALP = alkaline phosphatase; SGOT = serum glutamic oxaloacetic transaminase; SGPT = serum glutamic pyruvate transaminase

Parameter	Day 1	Day 2	Day 3	Day 4	Day 5	Day 7	Reference range
Hemoglobin (g/dL)	12.1	9.1	7.5	7.1	8.6	10.1	12–18
PCV (%)	38	26	21	21	31	25	36–54
MCV (fL)	101.6	101.9	102	100	92	96	80–96
Platelet count (k/µL)	304	201	231	206	120	110	150–450
Reticulocyte (%)	3	9.8	6.8	5.9	3.2	-	0.2–2
TLC (k/µL)	12	17.7	20.9	14.6	7.5	6.8	4–11
Sodium (mEq/L)	140	141		132	138	142	135–145
Potassium (mEq/L)	3.8	3.5	-	3.7	3.5	3.6	3.5–5.5
Chloride (mEq/L)	102	-	-	100	98	-	98–107
Urea (mg/dL)	29	-	-	40	45	-	15–45
Creatinine (mg/dL)	0.9	-	-	1.0	0.9	-	0.80–1.80
ALP (IU/L)	70	50	-	-	34	36	40–130
SGOT (IU/L)	57	115	-	-	44	45	5–40
SGPT (IU/L)	24	19	-	-	18	21	5–40
Total protein (g/dL)	7.7	6.9	-	-	-	6.6	6–8
Albumin (g/dL)	4.8	4.2	-	-	-	4.6	3.8–5.5
pH	7.45	7.427	-	7.466	-	7.415	7.35–7.45
HCO_3 _(mmol/L)	23.6	22.4	-	21.9	-	24	22–28
Lactate (mmol/L)	0.7	0.8	-	1.2	-	1.2	0.4–2.2
PCO_2 _(mmHg)	33.2	34.6	-	30.7	-	33.2	35–45
PO_2 _(mmHg)	77.9	82	-	85.9	-	92.9	75–105
SpO_2_ (%)	96.4	99.5	-	68.7	-	58.0	95–99
SpO_2_ RA	75	75	-	94	-	98	-
MetHb (%)	30	31	-	15	8	5	0.4–1.2

**Figure 2:**
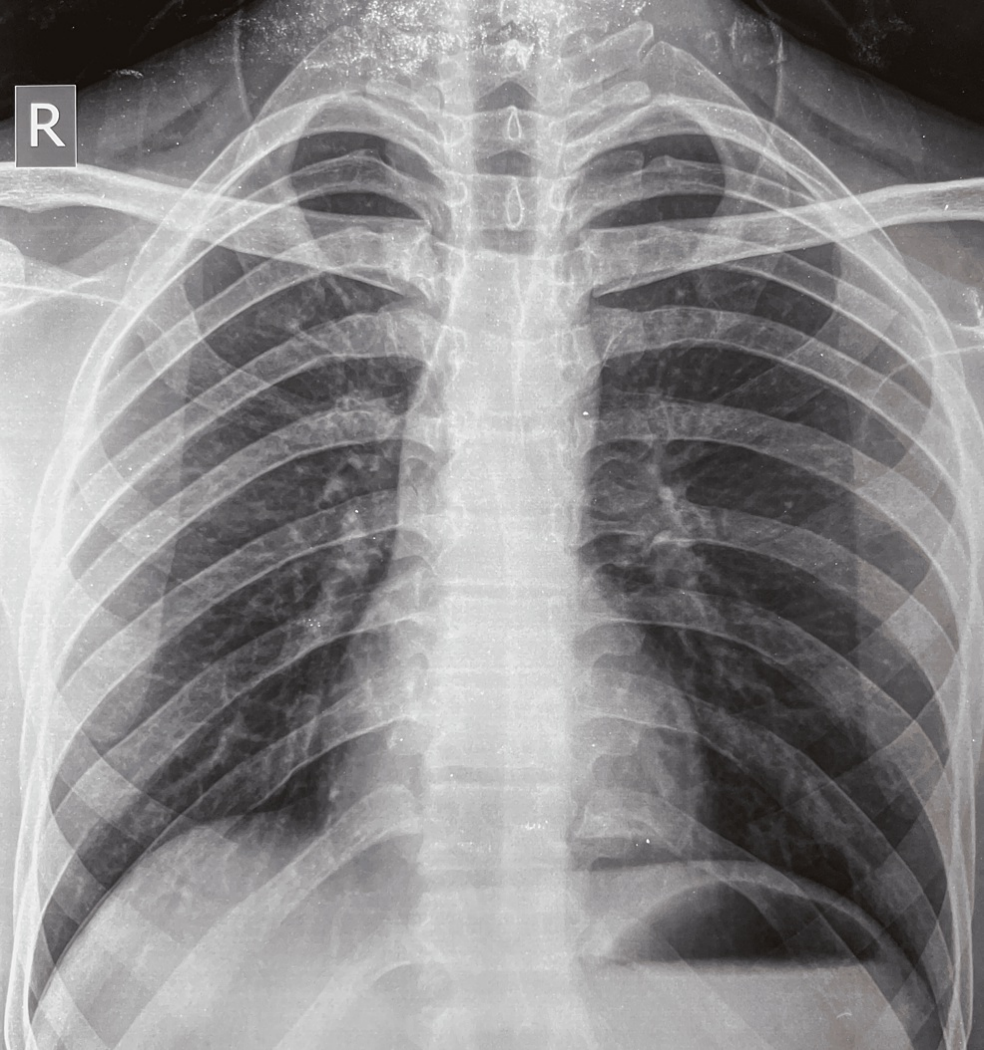
Chest X-ray of the patient showing clear lung fields.

Chest X-ray showed normal lung fields (Figure 2), and ultrasound of the abdomen was normal. There was no history of anemia, jaundice, or cola-colored urine and his two elder sisters were healthy. There was no history of any adverse drug reactions or allergies. A preliminary psychiatric assessment revealed a SAD PERSONS score of 4.

In view of the high concentration of MetHb, dark-colored urine (hemoglobinuria), high plasma hemoglobin, and hypoxia unresponsive to oxygen supplementation, the patient was administered 100 mg methylene blue intravenously over 10 minutes along with intravenous fluids.

However, the next day his condition started deteriorating. His jaundice and pallor worsened. In addition, the levels of transaminitis increased and his hemoglobin dropped by 4 g%. The urine was cola-colored. The renal function and creatinine phosphokinase, however, remained normal. His MetHb levels persistently remained elevated. Peripheral blood film (PBF) showed macrocytes with ovalocytes and occasional bite cells with an increased reticulocyte count of 9.8%. The possibility of hemolysis induced by methylene blue and underlying G6PDD was suspected. Any further administration of the antidote was avoided. We marked the patient as possible glucose 6 phosphate dehydrogenase deficient, and due to active hemolysis and increased reticulocyte count, it was decided to transfuse two units of packed RBCs and start the patient on intravenous vitamin C 2 g eight hourly. The sampling for the quantitative G6PDD test was deferred till the resolution of hemolysis as G6PD levels are highest in reticulocytes and young RBCs resulting in false-negative results [2,3].

The patient was managed conservatively with continued non-invasive oxygen therapy, adequate intravenous hydration, packed RBC transfusion guided by hemoglobin levels, and serial MetHb level monitoring. Daily CBC, reticulocyte count, and PBF as markers of RBC enzymatic activity status were noted. Reliance to fight oxidative stress was primarily on new RBCs in circulation with increased G6PD activity from packed RBC transfusion and his bone marrow response to acute hemolysis.

Over the course of the next seven days after methylene blue administration and 10 days after naphthalene ingestion, his MetHb levels dropped. Oxygen support was reduced to 2 L/minute via nasal cannula and was eventually stopped. His urine color normalized, and his general condition improved remarkably. He was stepped down from the medical intensive care unit to the high dependency unit and discharged in a hemodynamically stable condition with advice to follow up after five days with quantitative G6PD assay. Detailed information on red flag signs to watch out for was given to both the patient and his relatives along with a list of drugs to avoid in the future. On follow-up, his G6PD levels had reduced (2.75 IU/g hemoglobin), and the diagnosis was reaffirmed. He was sensitized to the matter and a psychiatry follow-up was arranged.

## Discussion

G6PDD is the most common enzyme deficiency disorder of RBCs. Approximately 400 to 500 million people in the world today are living with G6PDD. It is most common in tropical and subtropical regions with a prevalence as high as >60% in Kurdish Jews. Globally, people of Mediterranean, Puerto Rican, African, and Southeast Asian descent have a high probability of being G6PD deficient. Various studies among the Indian population have proposed its prevalence in India to be around 3%, 2.9%, and 1.9%. Some studies have mentioned G6PD Mediterranean to be the most common mutation, followed by Kerala-Kalyan, and Orissa, while other studies performed in malaria endemic zones have reported Orissa to be more common than the Mediterranean followed by Kerala-Kalyan. Jharkhand has the highest prevalence of G6PDD (6.3%), while Madhya Pradesh comes second (5.1%), followed by Gujrat (3.2%) [3,8,9].

Mostly, patients are diagnosed in infancy due to anemia or neonatal hyperbilirubinemia owing to hemolytic anemia or upon being treated for malaria with primaquine; however, the presence of anemia is variable depending on the severity of deficiency and lyonization. Based on the magnitude of enzyme deficiency and severity of hemolysis, the WHO has identified 200 variants of G6PDD. Types I and II have <10% enzyme activity, and type III has variable activity ranging between 10% and 60%. These are associated with chronic hemolytic anemia, intermittent hemolysis, and hemolysis only with significant oxidant stress, respectively. The most common variant in India is G6PD Mediterranean class II where patients can have neither anemia nor hemolysis with normal PBF except in the event of oxidant stress exposure [1-3,6].

The hepatic metabolism of naphthalene produces metabolites that overwhelm the body’s oxidative stress defense mechanisms in subjects with normal G6PD levels leading to hemolytic anemia. Anemia is more pronounced and associated with increased morbidity and mortality if the patient has G6PDD. The antidote for naphthalene intoxication is methylene blue, a thiazine dye, which can cause hemolytic anemia and is relatively contraindicated in patients with G6PDD. If MetHb levels are >30% or if levels are >20% in symptomatic patients with clinical features of hypoxemia or cerebral ischemia, an antidote is indicated. It requires nicotinamide adenine dinucleotide phosphate (NADPH) to reduce MetHb to oxyhemoglobin. As the generation of NADPH requires G6PD, methylene blue is not effective for G6PD-deficient patients [1-3].

Although there are point-of-care tests available that can be performed bedside and scored visually for G6PDD assessment before administering methylene blue, their use is limited by cost and restricted availability. On the other hand, as there is no defined threshold such as safe G6PD levels, avoidance of drugs known to cause severe oxidant stress is considered the best strategy [3]. Our patient did not have any history of hemolytic anemia or prolonged jaundice during infancy suggestive of G6PDD. We administered methylene blue in view of his severely high MetHb levels. Young RBCs have the highest level of G6PD activity, and it is expected that the hemolytic episode ends within a week of oxidant stress with a gradual reversal of anemia with the combined effect of packed RBC transfusion and reticulocyte response. Our patient showed significant improvement after the fifth day of the presentation. It is known that in the Mediterranean variant of G6PDD, the most common variant in India, the half-life of G6PD is shorter than other variants and recovery is gradual [2,3,8,9].

## Conclusions

An emergency physician regularly faces this clinical dogma as to whether or not to start treatment with methylene blue in naphthalene intoxication. When a history of hemolytic anemia or jaundice is lacking, the risk-benefit ratio favors cautious administration. The availability of bedside tests for G6PDD can theoretically prevent therapeutic misadventure. Vitamin C and NAC should be considered as alternatives in such cases.

## References

[1] Kuwada G, Murakami A, Glaser DW, Ingraham SE, Purohit PJ (2022). Mothball ingestion in the setting of G6PD deficiency causing severe hemolytic anemia, methemoglobinemia, and multiple organ failure in a toddler. Hawaii J Health Soc Welf.

[2] Pannu AK, Singla V (2020). Naphthalene toxicity in clinical practice. Curr Drug Metab.

[3] Glader B (2023). Diagnosis and management of glucose-6-phosphate dehydrogenase deficiency. UpToDate.

[4] Dubey D, Sharma VD, Pass SE, Sawhney A, Stüve O (2014). Para-dichlorobenzene toxicity - a review of potential neurotoxic manifestations. Ther Adv Neurol Disord.

[5] Weidman EK, Tsiouris AJ, Heier LA (2015). Toxic encephalopathy due to paradichlorobenzene toxicity: a case report and review of imaging characteristics. Clin Imaging.

[6] Ekambaram S, Chandan Kumar KM, Mahalingam V (2017). Acute kidney injury: a rare complication of mothball (naphthalene) poisoning. Saudi J Kidney Dis Transpl.

[7] Uthuman AA, Jayasinghe CS, Fernando AH (2019). Acute intravascular hemolysis due to naphthalene toxicity: a case report. J Med Case Rep.

[8] Kumar R, Singh MP, Mahapatra S (2020). Fine mapping of glucose 6 phosphate dehydrogenase (G6PD) deficiency in a rural malaria area of south west Odisha using the clinical, hematological and molecular approach. Mediterr J Hematol Infect Dis.

[9] Devendra R, Gupta V, Shanmugam R (2020). Prevalence and spectrum of mutations causing G6PD deficiency in Indian populations. Infect Genet Evol.

